# Blinatumomab Redirects Donor Lymphocytes against CD19^+^ Acute Lymphoblastic Leukemia without Relevant Bystander Alloreactivity after Haploidentical Hematopoietic Stem Cell Transplantation

**DOI:** 10.3390/ijms242216105

**Published:** 2023-11-09

**Authors:** Antonella Mancusi, Francesco Zorutti, Loredana Ruggeri, Samanta Bonato, Sara Tricarico, Tiziana Zei, Roberta Iacucci Ostini, Valerio Viglione, Rebecca Sembenico, Sofia Sciabolacci, Valeria Cardinali, Massimo Fabrizio Martelli, Cristina Mecucci, Alessandra Carotti, Maria Paola Martelli, Andrea Velardi, Antonio Pierini

**Affiliations:** 1 Division of Hematology and Clinical Immunology, Department of Medicine and Surgery, University of Perugia, 06129 Perugia, Italy; antonella.mancusi@unipg.it (A.M.);; 2 Division of Hematology and Clinical Immunology, Azienda Ospedaliera S. Maria Della Misericordia, 06132 Perugia, Italy

**Keywords:** acute lymphoblastic leukemia, allogeneic hematopoietic stem cell transplantation, immunotherapy, bispecific monoclonal antibody

## Abstract

Blinatumomab alone or with donor leukocyte infusions (DLI) has been used after allogeneic hematopoietic stem cell transplantation (HSCT) as a salvage therapy in relapsing patients with CD19^+^ hematological malignancies. It was effective in a fraction of them, with low incidence of Graft-versus-Host Disease (GvHD). Immunosuppressive drugs used as GvHD prophylaxis hinder T cell function and reduce the efficacy of the treatment. Because T cell-depleted haploidentical HSCT with donor regulatory and conventional T cells (Treg/Tcon haploidentical HSCT) does not require post-transplant immunosuppression, it is an ideal platform for the concomitant use of blinatumomab and DLI. However, the risk of GvHD is high because the donor is haploidentical. We treated two patients with CD19^+^ acute lymphoblastic leukemia (ALL) who had relapsed after Treg/Tcon haploidentical HSCT with blinatumomab and DLI. Despite the mismatch for one HLA haplotype, they did not develop GvHD and achieved complete remission with negative minimal residual disease. Consistently, we found that blinatumomab did not enhance T cell alloreactivity in vitro. Eventually, the two patients relapsed again because of their high disease risk. This study suggests that treatment with blinatumomab and DLI can be feasible to treat relapse after haploidentical transplantation, and its pre-emptive use should be considered to improve efficacy.

## 1. Introduction

Despite the recent introduction of new effective drugs, allogeneic hematopoietic stem cell transplantation (HSCT) is the main curative option for high-risk B cell acute lymphoblastic leukemia (ALL) [1]. It mainly relies on the Graft-versus-Tumor (GvT) effect exerted by donor alloreactive T cells. However, the same T cells can also attack normal tissues and cause Graft-versus-Host Disease (GvHD). Pharmacological immune suppression is widely used after transplant to prevent GvHD, but it inevitably counteracts the GvT effect and increases the risk of disease relapse. Indeed, whatever the transplant protocol, ALL relapse is still the main reason for failure after allogeneic HSCT in adults.

The TCR/CD19 bispecific antibody blinatumomab arms T cells against CD19^+^ cells, including malignant ones [2,3,4,5,6,7,8,9]. It has been used after allogeneic HSCT as a salvage therapy in relapsing patients with B cell malignancies. The treatment proved to be partially effective, with low incidence of GvHD [10,11,12,13]. To improve these results, studies are evaluating the use of blinatumomab in combination with donor leukocyte infusions (DLI) to treat relapsed B-ALL or B cell lymphoma [14,15,16,17]. Nevertheless, long-term immunosuppressive treatments might inhibit the function of donor T cells and thus antagonize the activity of blinatumomab in these patients.

Our institute has adopted a strategy of allogeneic HSCT that does not require post-transplant pharmacological immunosuppression, while allowing for the infusion of donor T cells to promote immunity against leukemia. Patients receive a “megadose” of hematopoietic stem cells from an HLA-haploidentical donor and adoptive immunotherapy with donor conventional T cells (Tcons) and regulatory T cells (Tregs) [18,19]. Donor Tregs control severe GvHD, despite the infusion of a high dose of HLA-haploidentical Tcons (1 × 10^6^/kg, which is almost 2 logs higher than the threshold for severe/lethal GvHD across a mismatch for an entire HLA haplotype) and the absence of any post-transplant immunosuppression. However, Tregs do not suppress the GvT effect, as chronic GvHD-free/relapse-free survival is above 70% in patients with acute myeloid leukemia [20]. An ongoing trial is investigating the outcomes of this approach in patients with ALL.

Because Treg/Tcon haploidentical HSCT does not require post-transplant immunosuppression, it represents an ideal platform for the combined employment of blinatumomab and DLI, even in an HLA-mismatched setting. Recently two patients with B cell malignancies who had undergone Treg/Tcon haploidentical HSCT and experienced disease relapse were treated with combined blinatumomab and DLI from their haploidentical transplant donors. Despite the mismatch for one HLA haplotype, patients did not develop GvHD and achieved complete remission with negative minimal residual disease. To explain such a clinical experience, we investigated how blinatumomab impacts donor T cell alloreactivity.

## 2. Results

**Figure 1:**
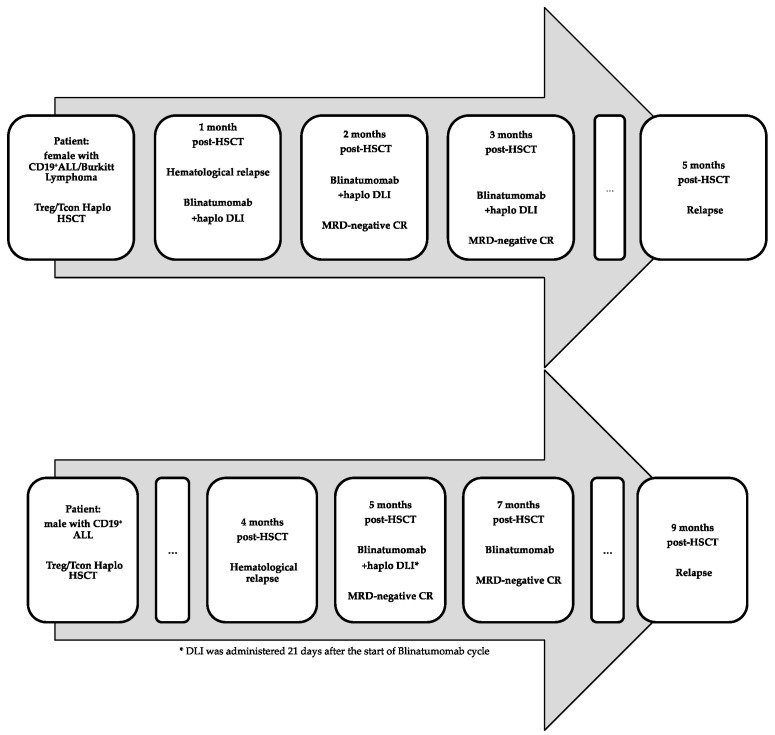
Timeline of patients’ treatments and disease course. ALL, acute lymphoblastic leukemia; Treg, regulatory T cell; Tcon, conventional T cell; Haplo, haploidentical; HSCT, hematopoietic stem cell transplantation; MRD, minimal residual disease; CR, complete remission.

Two patients with high-risk B cell malignancies underwent Treg/Tcon haploidentical HSCT with huge tumor burden and relapsed early after transplant. The first patient (woman, 50 years) had a mature CD19^+^ ALL/Burkitt lymphoma infiltrating her central nervous system and bone marrow at the time of transplant. The second patient (man, 42 years) had PBX1/E2A CD19^+^ ALL with positive minimal residual disease (FISH for PBX1/E2A: 0.9%). They were treated with blinatumomab plus standard unmanipulated DLIs from their haploidentical transplant donors at a maximum dose of 1 × 10^6^/kg. The treatment did not cause clinically relevant toxicity or acute or chronic GvHD, despite the relatively high dose of HLA-haploidentical DLIs that were infused early after transplant (the first infusion was given at 1 month in the first and at 5 months in the second patient) in the absence of immune suppression. Both patients achieved complete remission with negative minimal residual disease in the bone marrow. Eventually, both patients relapsed a few months after treatment in extramedullary sites. Details of the patients’ treatments and disease course are shown in Figure 1 and Table 1.

**Figure 2:**
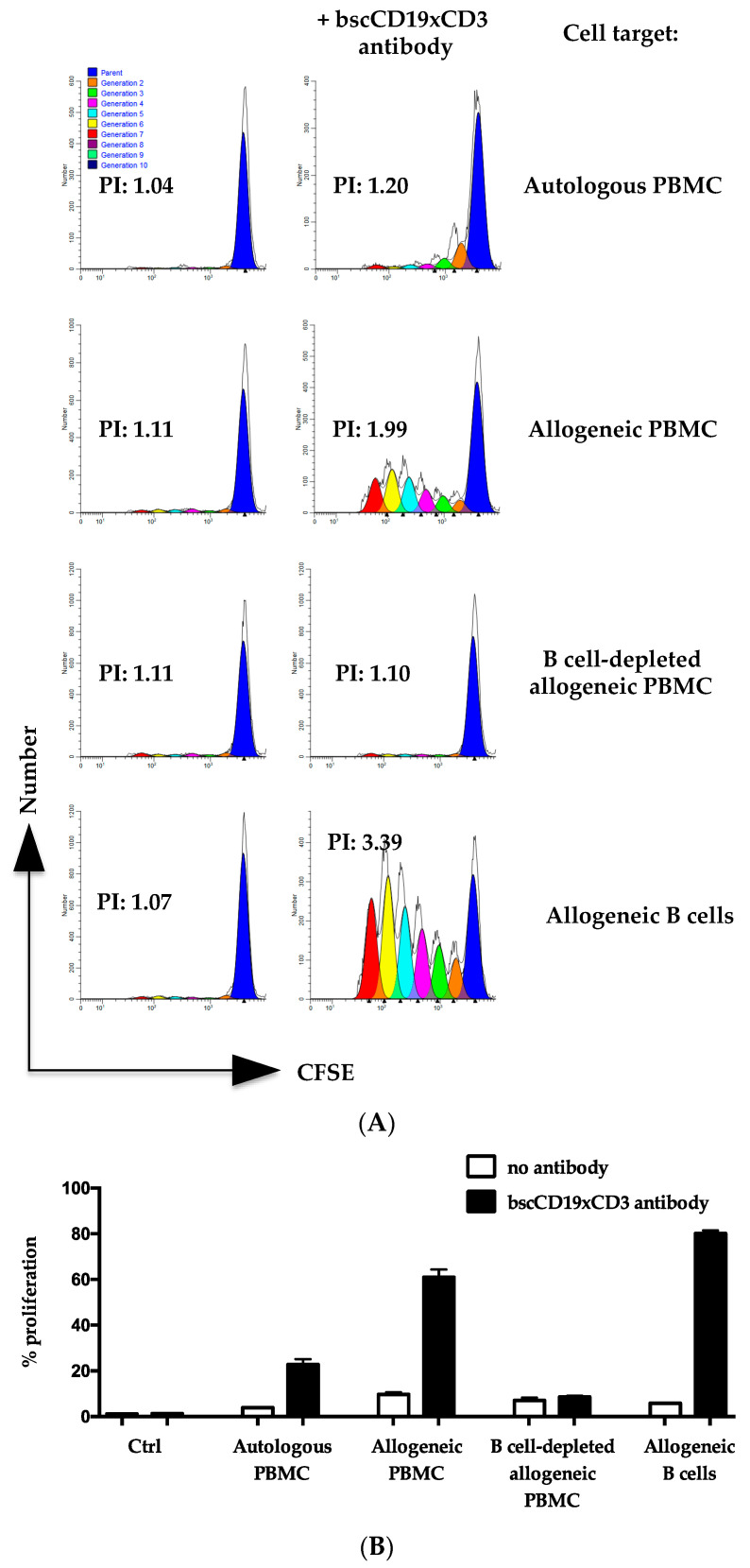
bscCD19xCD3 antibody did not enhance proliferation in response to B cell-depleted allogeneic targets. Proliferation of T cells after incubation with autologous PBMC or allogeneic PBMC, B cell-depleted PBMC and B cells, with or without a bscCD19xCD3 antibody, was evaluated via CFSE dilution assay. After 5 days, T cells were evaluated via flow cytometry to assess % of proliferating CFSE-low CD3^+^ T cells. (**A**) Representative analyses of cell division generations and proliferation indexes (PI) on CD3^+^CFSE^+^-gated cells. (**B**) % of proliferating CFSE-low CD3^+^ T cells in the absence of bscCD19xCD3 antibody or in the presence of bscCD19xCD3 antibody. Ctrl indicates a control without any cell target. Data are expressed as mean ± SD of replicates of the experiment shown in panel (**A**). Two independent experiments with effector and target cells isolated from two pairs of HLA-mismatched healthy subjects were performed for each panel. One representative experiment is shown. PBMC, peripheral blood mononuclear cells; bscCD19xCD3, anti-CD19/anti-CD3 single-chain bispecific antibody.

To understand why the patients did not develop GvHD despite a high dose of HLA-haploidentical DLI during blinatumomab treatment, we tested whether blinatumomab enhances T cell alloreactivity in in vitro assays. Because samples from the patients and their donors were not available, we used T cells and target peripheral blood mononuclear cells (PBMC) from HLA-mismatched unrelated healthy subjects. We compared T cell proliferation induced by an anti-CD19/anti-CD3 single-chain bispecific (bscCD19xCD3) antibody in response to autologous or allogeneic cell targets in a CFSE dilution assay. T cells were incubated with autologous or allogeneic PBMC, B cell-depleted PBMC, and B cells for 5 days. Figure 2 shows that the bscCD19xCD3 antibody enhanced T cell proliferation against both autologous and allogeneic PBMC, but this effect was abolished when the PBMC were depleted of B cells.

**Figure 3:**
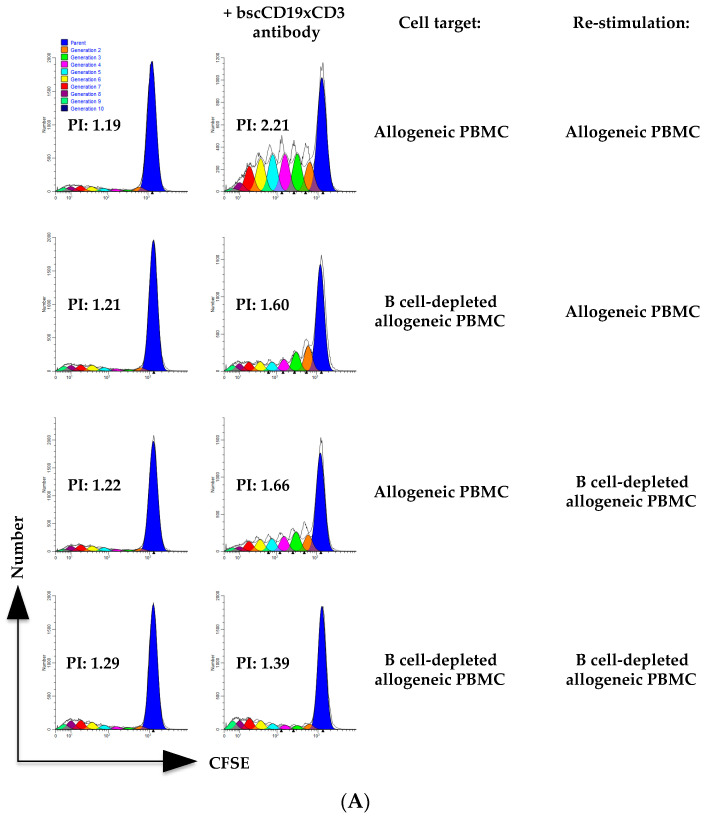

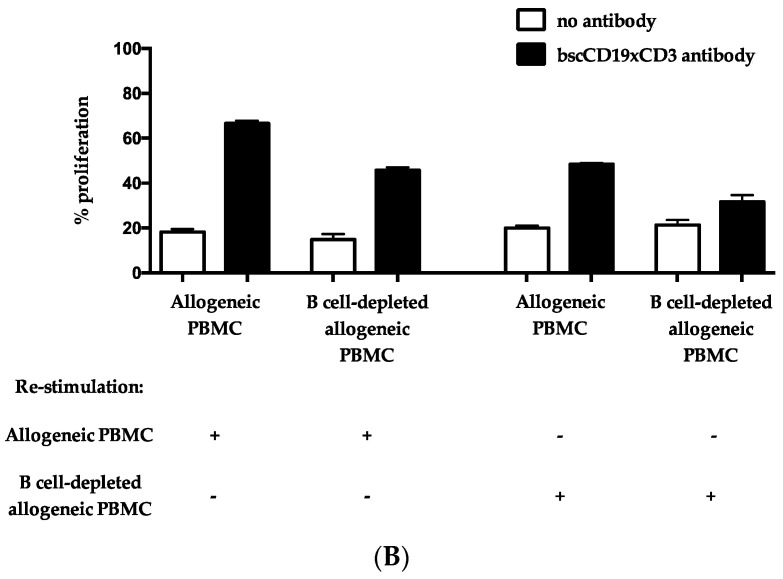
Re-stimulation with allogeneic target allowed for strong T cell proliferation only in the presence of B cells after bscCD19xCD3 antibody engagement. Proliferation of T cells after incubation with allogeneic PBMC or B cell-depleted PBMC with or without bscCD19xCD3 antibody was evaluated via CFSE dilution assay. On day 2, T cells were re-stimulated with allogeneic PBMC or B cell-depleted allogeneic PBMC. On day 5, T cells were evaluated via flow cytometry to assess % of proliferating CFSE-low CD3^+^ T cells. (**A**) Representative analyses of cell division generations and proliferation indexes (PI) on CD3^+^-gated cells. (**B**) % of proliferating CFSE-low CD3^+^ T cells in the absence of bscCD19xCD3 antibody or in the presence of bscCD19xCD3 antibody (+ sign indicates presence and − sign indicates absence of the indicated cell targets in the re-stimulation). Data are expressed as mean ± SD of replicates of the experiment shown in panel A. Two independent experiments with effector and target cells isolated from two pairs of HLA-mismatched healthy subjects were performed for each panel. One representative experiment is shown.

To further assess whether blinatumomab could enhance T cell alloreactivity, we performed the same experiment, re-stimulating T cells with the allogeneic cell targets, on day 2. After re-stimulation with B cell-depleted PBMC, the bscCD19xCD3 antibody enhanced T cell proliferation against B cell-depleted allogeneic cell targets less than in the presence of B cells (Figure 3).

**Figure 4:**
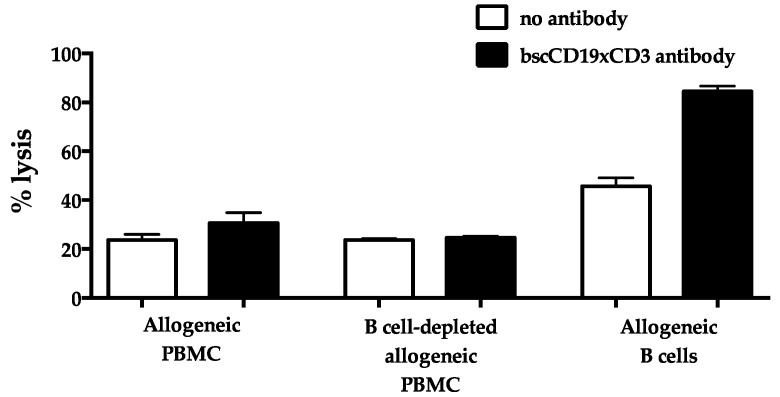
bscCD19xCD3 antibody did not enhance cytotoxicity in response to B cell-depleted allogeneic targets. Cytotoxicity mediated by T cells against allogeneic PBMC, B cell-depleted PBMC, and B cells in the absence of bscCD19xCD3 antibody (white bars) or in the presence of bscCD19xCD3 antibody (black bars) was evaluated via a ^51^Cr release assay. bscCD19xCD3 antibody enhanced cytotoxicity against allogeneic PBMC and B cells, but not B cell-depleted allogeneic PBMC. Data are expressed as mean ± SD of experimental replicates. Two independent experiments with effector and target cells isolated from two pairs of HLA-mismatched healthy subjects were performed. One representative experiment is shown.

Next, we evaluated the T cell-mediated cytotoxicity of the allogeneic cell targets induced by the bscCD19xCD3 antibody via a Chromium-51 (^51^Cr) release assay. T cells were expanded for 2 weeks with IL-2 and a purified anti-CD3 antibody in the presence of allogeneic PBMC, and then, re-incubated with ^51^Cr-labeled allogeneic cell targets with or without B cells for 4 h. Figure 4 shows that the addition of the bscCD19xCD3 antibody did increase cytotoxicity against allogeneic PBMC but not against allogeneic PBMC that were depleted of CD19^+^ cells. Thus, T cell cytotoxicity exerted by the bscCD19xCD3 antibody was also strictly dependent upon the presence of CD19^+^ cells in an allogeneic setting, and the antibody did not enhance T cell alloreactivity.

## 3. Discussion

We report the outcomes of two patients with CD19^+^ ALL who relapsed after Treg/Tcon haploidentical HSCT and were treated with concomitant blinatumomab and DLI. Both patients achieved complete remission without GvHD, despite the high dose of donor T cells infused across a mismatch for one HLA haplotype and the absence of any pharmacological immunosuppression. Consistently, we found that blinatumomab did not enhance T cell alloreactivity in vitro. Donor T cell engagement and activation by blinatumomab induced little proliferation in the absence of allogeneic CD19^+^ cells. One possible limitation of our experiments assessing proliferation could be the short time of T cell stimulation. In fact, when T cells were expanded for two weeks in the presence of allogeneic cell targets, T cell cytotoxicity was only enhanced by the presence of CD19^+^ cells and not by bystander HLA-mismatched PBMC. GvHD is caused by the expansion of donor-derived alloreactive T cell clones. Based on the present clinical and in vitro data, we can hypothesize that donor T cells efficiently expand but have limited alloreactive potential after TCR engagement by blinatumomab. Indeed, the antibody at a saturating concentration efficiently binds T cells, including alloreactive ones, and redirects them against CD19+ positive cells, while other allogeneic cells could be spared. Future studies are required to track TCR clonality [21] in a larger cohort of patients treated with blinatumomab after HSCT, in order to ascertain whether treatment with blinatumomab directs the whole repertoire against the target and prevents the expansions of T cell clones against the recipient HLA. At the time of haploidentical HSCT, the two patients also received adoptive immunotherapy with donor Tregs and Tcons. After relapse, they were treated with concomitant blinatumomab and DLI one month and five months after transplant. Although studies in mouse models suggest that Tregs exert their tolerogenic function early after transplants [22,23], a role for Tregs in the prevention of GvHD after treatment with blinatumomab and DLI cannot be excluded. Further experiments, such as tracking the TCR clonality of Tregs in patients receiving blinatumomab and DLI, could be useful to assess the contribution of Tregs in this setting.

Donor-derived T cells engineered to express chimeric antigen receptors (CAR-T cells) targeting CD19 [24,25] have been infused in small cohorts of patients with B cell malignancies who relapsed or with persistent disease after allogeneic HSCT, including a few patients transplanted from haploidentical donors. Complete remission was achieved in some patients with low incidence of GvHD [26,27]. Toxicity, primarily cytokine release syndrome and neurologic toxicity, and high costs currently limit its widespread use. Furthermore, CAR-T cell manufacturing is time-consuming and CAR-T cells are not immediately available at the moment of disease recurrence. The concomitant use of blinatumomab and DLI can be a safe and immediately available “CAR-T like” option to urgently treat post-transplant relapsed B cell ALL. At the same time, the probability that the treatment is effective long-term after disease relapse has occurred is very low. A recent study employed blinatumomab as a prophylactic treatment for relapse after HSCT. The study proved blinatumomab to be safe, with an acceptable rate of acute and chronic GvHD [28]. Our study supports the design of clinical trials to investigate the use of blinatumomab alone or in combination with DLIs after transplant, even in an HLA-haploidentical setting. Its early pre-emptive use should be considered to improve outcomes of B cell ALL patients at high risk of relapse.

## 4. Materials and Methods

### 4.1. Patients

This study was conducted according to the revised Helsinki Declaration. The presented data refer to two patients enrolled in a trial of Treg/Tcon haploidentical HSCT that was approved by the Umbria Regional Hospital Institutional Review Board. This trial was registered with the Umbria Region Institutional Review Board Public Registry under identification code 02/14 and public registry #2384/14, and at www.clinicaltrials.gov under #NCT03977103, accessed on 16 August 2023.

A detailed protocol of Treg/Tcon haploidentical transplantation was published previously [15,16,17]. Briefly, the conditioning regimen included total body irradiation, thiotepa, fludarabine, and cyclophosphamide. Patients received an infusion of 2 × 10^6^/kg donor regulatory T cells on day-4, followed by 1 × 10^6^/kg donor conventional T cells on day-1 (isolated from unstimulated donor apheresis) and a mean of 10.65 × 10^6^/kg purified CD34^+^ hematopoietic progenitor cells on day 0. No pharmacological GvHD prophylaxis was administered post-transplantation.

To treat post-transplant CD19^+^ ALL relapse, patients received the indicated cycles of blinatumomab (©Amgen, Thousand Oaks, CA, USA), 9 μg/day for the first 7 days and 28 μg/day thereafter via continuous intravenous infusion over 4 weeks. During treatment with blinatumomab, they also received standard unmanipulated DLI from their haploidentical transplant donors (up to 1 × 10^6^/kg, isolated from unstimulated donor apheresis).

### 4.2. CFSE Dilution Assay

T cells and autologous or allogeneic target cells were obtained from PBMC of healthy volunteers. Allogeneic B cells and B cell-depleted PBMC were isolated via immunomagnetic selection with human CD20 microbeads (Miltenyi Biotec, Bergisch Gladbach, Germany). T cells were isolated from B cell-depleted PBMC via negative selection with human CD14 and CD16 microbeads (Miltenyi Biotec, Bergisch Gladbach, Germany). Additional negative selection with human CD19 microbeads was performed on B cell-depleted PBMC to remove residual B cells (Miltenyi Biotec, Bergisch Gladbach, Germany). Autologous or allogeneic target cells were irradiated at 30 Gy. T cells were labeled with 5 µM CFSE (Life Technologies Corp., Carlsbad, CA, USA), and 1 × 10^5^ T cells were incubated in triplicate in the presence of medium alone or 2 × 10^5^ autologous or allogeneic target cells, with or without 10 ng/mL of bscCD19xCD3 antibody (Blinatumomab Biosimilar, ichorbio, Wantage, UK). After 5 days, cells were labeled with an anti-CD3 PE-Vio770 antibody (clone REA613, Miltenyi Biotec, Bergisch Gladbach, Germany) and evaluated via flow cytometry to assess the % of proliferating CFSE-low CD3^+^ T cells using FACSCanto and FACSDiva Software version 6.1.3 (BD Biosciences, Franklin Lakes, NJ, USA) and ModFit LT software version 6.0 (Verity Software House, Topsham, ME, USA). The same experiment was performed, adding 2 × 10^5^ allogeneic target cells on day 2 of the initial incubation, in order to re-stimulate CFSE-labeled T cells. In this experiment, target cells were also depleted of CD3^+^ T cells with human CD4 and CD8 microbeads (Miltenyi Biotec, Bergisch Gladbach, Germany), as they could overlap with highly proliferating effector T cells with a very low intensity of CFSE.

### 4.3. ^51^Cr Release Assay

T cells and allogeneic target cells were obtained from PBMC of healthy volunteers and isolated as described above. T cells were stimulated for 2 weeks in a 1:2 ratio with irradiated allogeneic PBMC in the presence of 125 UI/mL IL-2 (Miltenyi Biotec, Bergisch Gladbach, Germany) and 1 μg/mL purified anti-CD3 antibody (clone OKT3, Life Technologies Corp., Carlsbad, CA, USA). Then, they were incubated in triplicate in a 25:1 ratio with 2 × 10^4^ allogeneic target cells labeled with ^51^Cr radionuclide (Perkin Elmer, Waltham, MA, USA) in the presence or in the absence of 0.5 μg/mL of bscCD19xCD3 antibody for 4 h. Counting was performed using a Micro Beta liquid scintillation counter (Perkin Elmer, Waltham, MA, USA). The % of specific lysis was evaluated as follows: [(experimental release-spontaneous release)/(maximum release-spontaneous release)] × 100.

## Figures and Tables

**Table 1:** Details of patients’ treatment with blinatumomab and DLI.

Patient	Diagnosis	Time of Post-HSCT Relapse	Blinatumomab	DLIs (Dose)	CRS	Neurotoxicity	Acute or Chronic GvHD	Response
Female, 50 years	CD19^+^ ALL/Burkitt lymphoma	1 month	3 cycles	3 (0.5–1 × 10^6^/Kg)	No	No	No	MRD^-^ CR
Male, 42 years	CD19^+^ ALL	4 months	2 cycles	1 (0.5 × 10^6^/Kg)	No	Yes §	No	MRD^-^ CR

§, Confusion that required temporary suspension of the treatment and brief use of steroids. ALL, acute lymphoblastic leukemia; CRS, cytokine release syndrome; MRD- CR, complete remission with negative minimal residual disease.

## Data Availability

The data presented in this study are available in the manuscript.
